# Natural Nitrogen-Doped Carbon Dots Obtained from Hydrothermal Carbonization of Chebulic Myrobalan and Their Sensing Ability toward Heavy Metal Ions

**DOI:** 10.3390/s23020787

**Published:** 2023-01-10

**Authors:** Raji Atchudan, Suguna Perumal, Thomas Nesakumar Jebakumar Immanuel Edison, Ashok K. Sundramoorthy, Rajangam Vinodh, Sambasivam Sangaraju, Somasundaram Chandra Kishore, Yong Rok Lee

**Affiliations:** 1 School of Chemical Engineering, Yeungnam University, Gyeongsan 38541, Republic of Korea; 2 Department of Chemistry, Sejong University, Seoul 143747, Republic of Korea; 3 Department of Prosthodontics, Saveetha Dental College and Hospitals, Saveetha Institute of Medical and Technical Sciences, Poonamallee High Road, Velappanchavadi, Chennai 600077, Tamil Nadu, India; 4 Green Hydrogen Lab (GH2Lab), Institute for Hydrogen Research (IHR), Université du Québec à Trois-Rivières (UQTR), 3351 Boulevard des Forges, Trois-Rivières, QC G9A 5H7, Canada; 5 National Water and Energy Center, United Arab Emirates University, Al Ain 15551, United Arab Emirates; 6 Saveetha School of Engineering, Department of Biomedical Engineering, Saveetha Institute of Medical and Technical Sciences, Saveetha Nagar, Chennai 602105, Tamil Nadu, India

**Keywords:** Chebulic Myrobalan, hydrothermal carbonization, N-doped carbon dot, fluorometric sensor, heavy metal ion, aqueous medium

## Abstract

Chebulic Myrobalan is the main ingredient in the Ayurvedic formulation Triphala, which is used for kidney and liver dysfunctions. Herein, natural nitrogen-doped carbon dots (NN-CDs) were prepared from the hydrothermal carbonization of Chebulic Myrobalan and were demonstrated to sense heavy metal ions in an aqueous medium. Briefly, the NN-CDs were developed from Chebulic Myrobalan by a single-step hydrothermal carbonization approach under a mild temperature (200 °C) without any capping and passivation agents. They were then thoroughly characterized to confirm their structural and optical properties. The resulting NN-CDs had small particles (average diameter: 2.5 ± 0.5 nm) with a narrow size distribution (1–4 nm) and a relatable degree of graphitization. They possessed bright and durable fluorescence with excitation-dependent emission behaviors. Further, the as-synthesized NN-CDs were a good fluorometric sensor for the detection of heavy metal ions in an aqueous medium. The NN-CDs showed sensitive and selective sensing platforms for Fe^3+^ ions; the detection limit was calculated to be 0.86 μM in the dynamic range of 5–25 μM of the ferric (Fe^3+^) ion concentration. Moreover, these NN-CDs could expand their application as a potential candidate for biomedical applications and offer a new method of hydrothermally carbonizing waste biomass.

## 1. Introduction

Carbon dots/carbon quantum dots are a new class of the most recent nanocarbon materials. They were first discovered by Xu et al. in 2004 [1] during the purification of single-wall carbon nanotubes (SWCNTs). In this, SWCNTs were synthesized through the arc-discharge method. Carbon dots belong to zero-dimensional materials with quasi-spherical shapes, and their sizes are below 10 nm [2,3,4]. They have had considerable attention in recent years due to their attractive characteristics such as excellent water dispersibility, multicolor fluorescence emissions, prolonged colloidal durability in aqueous media, excellent photostability, good biocompatibility, cost-effective production, unique chemical inertness, and tunable surface functionalities [5,6,7,8,9]. Moreover, the synthesis process of carbon dots does not involve any hazardous heavy metals; hence, it is environmentally friendly. Due to the outstanding physicochemical, optical, and biological characteristics, carbon dots have had widespread attention from several important sectors such as sensor/biosensor, catalysis, biomedical, wastewater purification, optoelectronic device, and anti-counterfeiting issues [10,11,12,13]. Several methods have been proposed for synthesizing carbon dots such as microwave heating, laser ablation, arc-discharge, electrochemical oxidation, plasma treatments, combustion/thermal pyrolysis, solvothermal heating, and hydrothermal carbonization methods [14,15,16,17,18]. Compared with other methods, the hydrothermal carbonization route with natural precursors shows significant advantages such as a simple setup, being green/clean and environmentally friendly, and a cost-effective design [19,20,21].

On the other hand, metal ions are essential to sustain the health of humans because several biological functions depend upon the presence of metal ions, and several enzymes require those elements for their catalytic reaction [22,23]. Among all the necessary metal ions for regular biological functions in the human system, iron (Fe^3+^) plays a momentous role in many physiological processes such as oxygen transport, deoxyribonucleic acid synthesis, ribonucleic acid synthesis, enzyme catalysis, energy production, and cellular respiration/metabolism [24,25]. Notably, it has a well-known function in the blood protein hemoglobin of human blood (a key component of red blood cells) [19,26]. Iron intake mostly comes from food such as vegetables, fruit, eggs, and milk. The excess and deficiency of iron in the human body can cause physiological damage and biological disorders, which perhaps result in several serious diseases such as anemia, diabetes, arthritis, hemochromatosis, a lower immunity, a lower blood pressure, liver cirrhosis, heart disease, kidney failure, and even cancer [5,10,27]. The level of iron in the body needs to be monitored and maintained at an appropriate range for sustaining the function of our body. Thus, detecting iron in potable water and the water environment is essential. A few standard methods such as electrochemistry, inductively coupled plasma mass spectrometry, and atomic absorption spectrometers are available to detect Fe^3+^ ions [28]. The fluorescent sensor method has gained increasing attention for the sensing (detection) of metal (heavy) ions due to its simplicity and high selectivity as well as its sensitivity, great spatial resolution, and possibility for practical, quick monitoring [29,30,31]. Nevertheless, such detection methods require expensive instruments and take a long time to process. Hence, developing a simple and cost-effective detection method with high sensitivity and selectivity is necessary for finding Fe^3+^ ions.

Herein, the preparation of natural nitrogen-doped carbon dots (NN-CDs) from low-cost Chebulic Myrobalan as a green precursor for both nitrogen and carbon by a one-step hydrothermal-assisted carbonization approach was explored and used for the recognition of Fe^3+^ ions. There have been reports of pleiotropic effects from the dried fruit extract of Chebulic Myrobalan, also called *Terminalia chebula* Fructus Retz. (*Combretaceae*), which is regarded as the “King of Medicine” in Tibet and is considered to be a universal panacea in traditional Indian medicine [32]. It has been demonstrated to have anticancer, antioxidant, antimicrobial, anti-anaphylactic, and adaptogenic activities in the literature and is described as a laxative, diuretic, and cardiotonic substance [33]. Numerous phenolic compounds, glycosides (i.e., triterpene arjunglucoside I, the chebulosides I and II, and arjungenin), coumarin ligated with gallic acids (chebulin), ellagic acid, chebulinic acid, 2,4-chebulyl-D-glucopyranose, ethyl gallate, punicalagin, and terflavin A have been reported to be extracted from Chebulic Myrobalan [33]. Structural characterizations were employed for the prepared NN-CDs to determine their morphology, particle size, and structural properties. The optical studies of the prepared NN-CDs, including the ultraviolet-visible (UV-vis) absorption spectroscopy and fluorescence excitation/emission spectroscopy, were examined to resolve the fluorescence behavior and photostability. Later, the successfully prepared NN-CDs were used as fluorescent sensors for the quantitative detection of Fe^3+^ ions by a fluorescence quenching mechanism.

## 2. Results and Discussion

**Figure 1:**
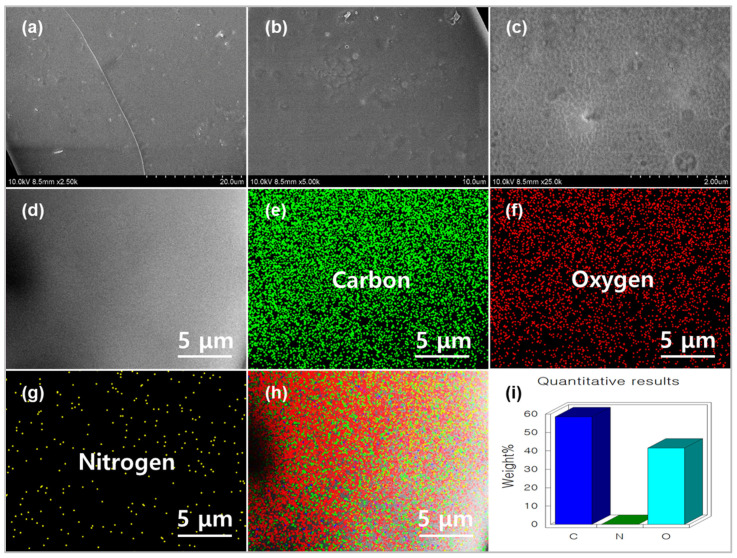
(**a**–**d**) FE-SEM images with different magnifications of prepared natural nitrogen-doped carbon dots. Elemental mapping of (**e**) carbon, (**f**) oxygen, (**g**) nitrogen, and (**h**) overlapping of all the elements of prepared natural nitrogen-doped carbon dots. (**i**) Quantitative results of presented elements in the prepared natural nitrogen-doped carbon dots.

The surface morphologies and chemical/elemental compositions of the synthesized NN-CDs were revealed by field emission-scanning electron microscopy and an energy-dispersive X-ray (FE-SEM-EDX) elemental mapping analysis. As depicted in Figure 1a–d, the accumulation of the small size of the NN-CDs resulted in a smooth surface morphology. This accumulation may have been because of the coalescence of the NN-CDs due to the evaporation of the solvent during the sample preparation for the FE-SEM studies. Further, the elemental compositions of the prepared NN-CDs were revealed via an EDX elemental mapping analysis. The EDX mapping (Figure 1e–g) displayed that the NN-CDs exhibited carbon (green), oxygen (red), and nitrogen (yellow) elements. The elemental overlay image of the NN-CDs (Figure 1h) confirmed that the heteroatoms (functionalities) such as oxygen and nitrogen were consistently distributed over the carbon skeleton of the NN-CDs. The EDX investigation of the prepared NN-CDs proved the existence of carbon, oxygen, and nitrogen in the prepared NN-CDs (Figure S1). Apart from these elemental peaks, minor peaks appeared around the energy of 2 keV in the EDX spectrum, which was related to the silicon and platinum elements that originated from the silicon wafer and ohmic contact coating during the sample preparation for the FE-SEM-EDX analysis. The elemental percentages of carbon, oxygen, and nitrogen were approximately 63, 34, and 3, respectively, obtained from the prepared NN-CDs; these were more evident in the bar chart (Figure 1i) for the EDX results. From these results, we concluded that the NN-CDs had carbon, oxygen, and nitrogen elements.

**Figure 2:**
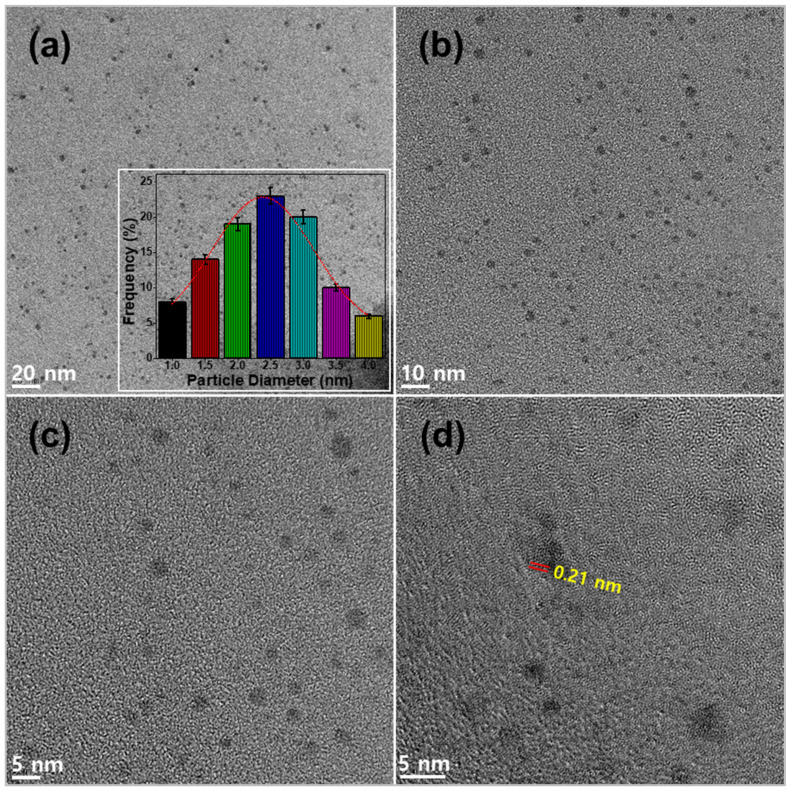
TEM/HRTEM images with different magnifications of prepared natural nitrogen-doped carbon dots.

Furthermore, the transmission electron microscopy/high-resolution transmission electron microscopy (TEM/HRTEM) analyses confirmed the morphology, particle size, and degree of the crystalline/graphitic nature of the prepared NN-CDs. The TEM images (Figure 2a,b) displayed that the NN-CDs were predominantly spherical in shape with excellent monodispersity. The size distribution of the prepared NN-CDs was in a range between 1 nm and 4 nm with an average diameter of 2.5 ± 0.5 nm (inset of Figure 2a). Figure 2c,d are the HRTEM images of the NN-CDs, showing the well-resolved lattice fringes in the core of the NN-CDs with a d-spacing of 0.21 nm corresponding with the (1 0 0) in-plane lattice fringes of typical carbon/graphene, which suggested the crystalline nature of the prepared NN-CDs [34].

**Figure 3:**
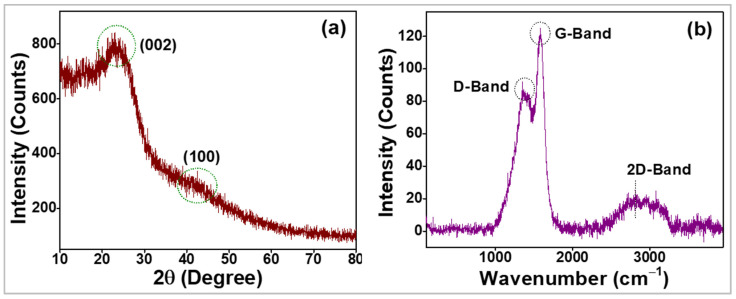
(**a**) XRD pattern and (**b**) Raman spectrum of prepared natural nitrogen-doped carbon dots.

The XRD pattern and Raman spectrum were performed to examine the crystalline/graphitic structure of the prepared NN-CDs. Figure 3a depicts the XRD pattern of the NN-CDs, revealing a broad peak around 2θ = 24.5°, which corresponded with the typical (0 0 2) diffraction peak of standard carbon/graphene [35,36]. The calculated d-spacing value of the NN-CDs was 0.36 nm, which was greater than that of a standard graphene interlayer spacing (0.34 nm, d-spacing), along with the broadness character. The larger interlayer spacing and peak broadening were associated with an amorphous carbon phase and a moderately graphitized structure [37]. The higher interlayer spacing might also have been due to the presence of various functional groups on the surface and the edges of the prepared NN-CDs [38]. Hence, the prepared NN-CDs displayed a moderate degree of graphitization/crystallinity. A small shoulder peak around 42.5° was associated with the C (1 0 0) Bragg reflection of typical graphene; the calculated interlayer spacing was 0.21 nm. Furthermore, the Raman spectrum was recorded to determine the graphitization degree of the NN-CDs. The G-band at 1590 cm^−1^ was related to the vibration of the sp^2^ hybridized carbon phase associated with ordered/crystalline carbon whereas the D-band at 1390 cm^−1^ was associated with the vibrations of the sp^3^ hybridized carbon phase originating from disordered/amorphous carbon [39]. The D-band to G-band intensity ratio (I_D_/I_G_) is an efficient indicator of the determination of the graphitization/crystallinity of carbon-based materials [40]. The I_D_/I_G_ ratio of the NN-CDs was around 0.73, indicating that the prepared NN-CDs had a moderate degree of graphitization. A broad 2D band of the NN-CDs was observed at 2800 cm^−1^, suggesting that the NN-CDs exhibited a few-layer graphene structure consistent with the HRTEM results [41]. Overall, the results from the HRTEM, XRD, and Raman studies revealed the moderate graphitic nature of the NN-CDs.

Attenuated total reflection Fourier-transform infrared (ATR-FTIR) spectroscopy was employed in this study to help to ascertain and examine the possible bonds (functional moieties) present on the surface and edges of the prepared NN-CDs and is displayed in Figure S2. The ATR-FTIR spectrum of the prepared NN-CDs showed several peaks related to their surface bonds. The broad absorption peak spanning from 3500 to 3000 cm^−1^ was ascribed to the stretching vibrations of the O–H bond, which may have originated from the source of the starting materials and physically adsorbed water molecules on the surface/edges of the NN-CDs [42]. The absorption band between 3500 and 3000 cm^−1^ might have been caused by the stretching vibrations of the N–H bond [43,44]. These presented bonds were perhaps accountable for imparting the hydrophilicity and consequent excellent water dispersibility of the NN-CDs. The stretching vibration of minor C–H peaks at 2952 cm^−1^ in the ATR-FTIR spectrum of the NN-CDs specified that the NN-CDs were composed of few hydrocarbons. On the other hand, the ATR-FTIR spectrum of the NN-CDs had significant peaks at 1701, 1600, 1475, 1312, 1195, and 1090–960 cm^−1^ corresponding with the C=O stretching, C=C stretching, C–N stretching, O–H bending, C–OH stretching, and C–O–C stretching modes [45,46]. The peak at 770 cm^−1^ could be ascribed to the out-plane –CH_2_ stretching and N–H bending vibrations of the aromatic carbon structure [47]. The existence of a C=C peak signposted that the NN-CDs had a graphitic structure whereas the O–H, N–H, C=O, C–N, and C–H vibration modes suggested that the NN-CDs had amine, hydroxyl, and carboxylic surface moieties.

**Figure 4:**
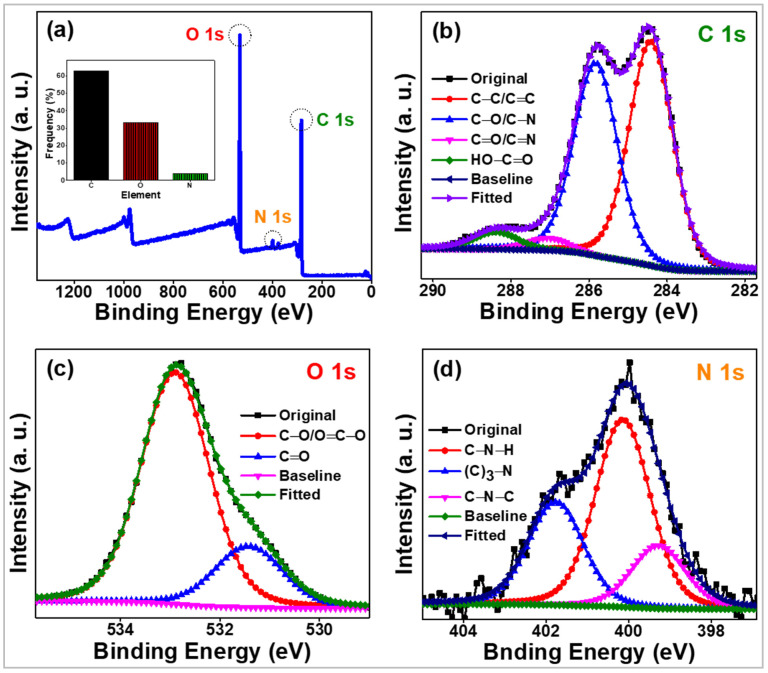
XPS spectra of the prepared natural nitrogen-doped carbon dots: (**a**) survey scan spectrum, (**b**) high-resolution C 1s electron level, (**c**) high-resolution O 1s electron level, and (**d**) high-resolution N 1s electron level. Inset (**a**): bar chart of the atomic percentages of existing elements in the prepared natural nitrogen-doped carbon dots.

An X-ray photoelectron spectroscopy (XPS) analysis of the NN-CDs also helped us to portray and examine the possible chemical structure of the moieties attached to the surface/edges of the prepared NN-CDs. Figure 4a depicts the survey spectrum of the prepared NN-CDs. The spectrum displayed that the NN-CDs were mainly composed of a few primary elements such as carbon, oxygen, and nitrogen, with atomic ratios of 63, 33, and 4%, respectively (inset Figure 4a). The core level C 1s spectra (Figure 4b) displayed four peaks that appeared at 284.4, 285.8, 287.1, and 288.4 eV, originating from the carbon in the states of the C–C/C=C (sp^3^/sp^2^), C–N/C–OH/C–O–C, C=O/C=N, and O=C–OH groups in the NN-CDs, respectively [48,49]. The deconvolution of the O 1s level of the NN-CDs (Figure 4c) showed two peaks at 531.4 and 532.8 eV that were responsible for the presence of the C=O and C–OH/C–O–C/O=C–OH groups in the NN-CDs, respectively [50,51,52]. The N 1s spectra (Figure 4d) exhibited three peaks at 399.3, 400.2, and 401.8 eV and were ascribed to nitrogen in the form of pyridinic nitrogen (C–N–C), pyrrolic nitrogen (C–N–H), and quaternary amine/graphitic nitrogen (C_3_–N) groups in the NN-CDs, respectively [53,54]. The pyrrolic nitrogen possibly increased the electronic cloud density on the surface/edges of the NN-CDs, resulting in an enhanced fluorescence efficiency [48]. This XPS result was in good agreement with the ATR-FITR analysis. Overall, these results confirmed that the prepared NN-CDs possessed various functional groups such as carbonyl (C=O), hydroxy (–OH), carboxyl (O–C=O), and amine (N–H) groups. More than one out of every three carbon atoms was functionalized with oxygen, which showed that the NN-CDs exhibited a high degree of functionalization. Moreover, the high degree of functionalization with excellent hydrophilicity was the possible reason that the prepared NN-CDs possessed excellent water dispersibility and fluorescence properties as well as long-term colloidal stability in an aqueous medium [50,55].

**Figure 5:**
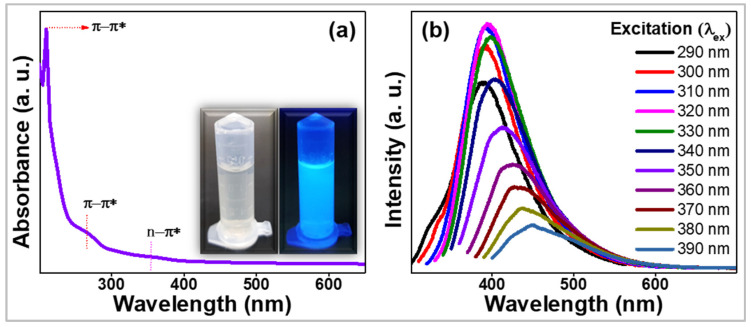
(**a**) UV-vis absorption spectrum and (**b**) fluorescence emission spectrum upon varying the excitation of prepared natural nitrogen-doped carbon dots. Inset (**a**): digital photographs of aqueous solution of natural nitrogen-doped carbon dots under daylight (left) and 365 nm UV light (right).

UV-vis absorption spectroscopy and fluorescence excitation/emission spectroscopy were used to examine the optical properties of the NN-CDs. As shown in Figure 5a, a sharp and distinct absorption peak in the ultraviolet region with maximum absorption at 210 nm confirmed the π–π* transition of the aromatic sp^2^ domains in the NN-CDs. The shoulder bumps were centered at approximately 265 and 350 nm, resulting from the π–π* and n–π* transitions of the C=C (aromatic sp^2^ domains) and C=O/C=N (heteroatom-containing bonds) bands in the prepared NN-CDs, respectively [56,57,58]. The inset of Figure 5a shows the digital photographic images of the aqueous solution of the prepared NN-CDs. When the solution of the NN-CDs was exposed to daylight (left) and 365 nm UV light (right), it emitted a pale yellow color and a bright cyan-blue color, respectively. The excitation and emission fluorescence spectra of the prepared NN-CDs in the aqueous solution are shown in Figure S3a. The most substantial fluorescence emission peak was centered at 395 nm and showed a maximum excitation wavelength at 320 nm in the fluorescence spectra of the NN-CDs. The fluorescence spectra of the NN-CDs in Figure 5b showed that the maximum fluorescence emission wavelength of the NN-CDs reached a peak at 395 nm with an excitation wavelength of 320 nm. The fluorescence emission wavelength shifted from 387 to 450 nm when the excitation wavelength changed from 290 to 390 nm at an increment of 10 nm. During this, the fluorescence emission intensity gradually increased until it reached an excitation wavelength of 320 nm; beyond that, a gradual decrement was followed by the increment of the excitation wavelength. Overall, the fluorescence emission peaks turned to redshift whilst increasing the excitation wavelength. The redshift of the fluorescence emission of the NN-CDs was more evident when the fluorescence emission intensity was normalized at different excitation wavelengths; the corresponding excitation-dependent emission normalized spectrum is shown in Figure S3b. The result revealed that the NN-CDs had an excitation-dependent fluorescence emission, which was attributed to surface/edge defects, the quantum confinement effect, and various particle sizes [59,60,61]. The quantum yield of the NN-CDs was calculated to be around 15% with an excitation wavelength of 320 nm by a standard fluorescence quantum yield measurement (Equation (S1)) [3] where quinine sulfate was used as the reference.

In order to evaluate the photostability of the prepared NN-CDs, the aqueous solution of the NN-CDs was subjected to 365 nm UV illumination from 0 to 100 min; the fluorescence intensities were measured before and after the illumination. The photostability of the prepared fluorescent nanomaterials (NN-CDs) was essential for their practical/real-time applications. The fluorescence emission spectra of the aqueous solution of the NN-CDs at 0 and 100 min UV illumination are shown in Figure S4. The spectra depicted that there were no apparent changes (only less than a 5% change in its fluorescence emission) in the fluorescence intensities of the aqueous solution of the NN-CDs even after 100 min of continuous irradiation, suggesting that the NN-CDs possessed an excellent anti-photobleaching behavior (photostability) [62]. Photos of the aqueous solution of the NN-CDs were taken before and after 100 min UV illumination under the 365 nm UV light, which is shown in the inset of Figure S4. The digital photographs of the aqueous solution of the NN-CDs displayed insignificant changes in the fluorescence emission even after 100 min of continuous irradiation (the fluorescence brightness was identical at both 0 and 100 min). This also supported the anti-photobleaching of the prepared NN-CDs.

**Figure 6:**
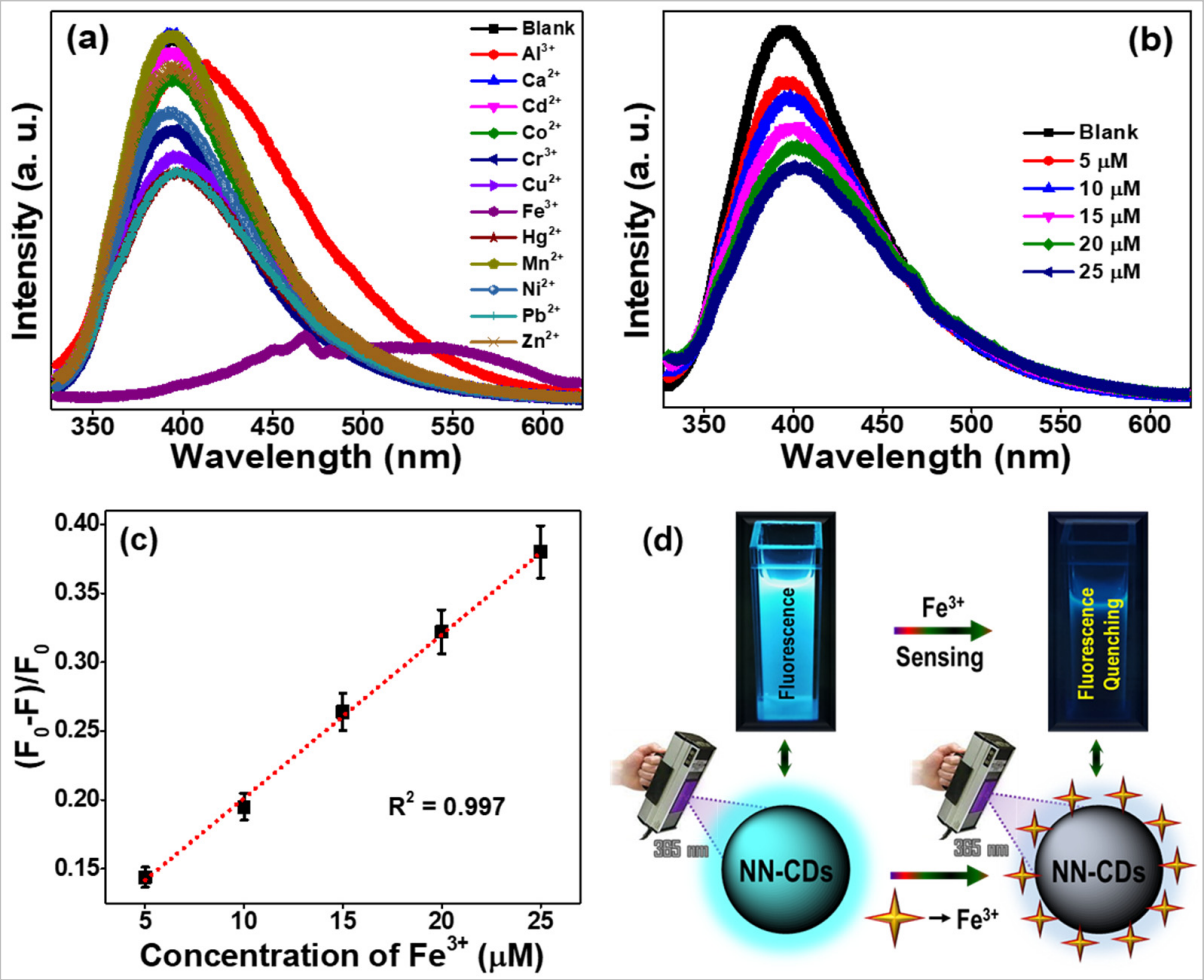
(**a**) Fluorescence emission changes of the aqueous solution of NN-CDs with the addition of various metal ions at a concentration of 500 µM. (**b**) Fluorescence intensity of the aqueous solution of NN-CDs with different concentrations of Fe^3+^ ions (0 (blank), 5, 10, 15, 20, and 25 µM). (**c**) The Stern–Volmer plot of the aqueous solution of NN-CDs with increasing concentrations of Fe^3+^ ions. (**d**) Digital photographs of aqueous NN-CD suspension before and after adding Fe^3+^ ions under 365 nm UV light illumination (upper) and a schematic representation of the fluorescence emission quenching triggered by the chelation of Fe^3+^ ions.

After the careful characterization of the NN-CDs using various methods, the NN-CDs were explored as a fluorescence probe for detecting heavy metal ions because an excess/deficiency of heavy metal ions is dangerous to living things, especially humans. Hence, the detection of metal ions in ecological systems is essential for society. The prepared NN-CDs had high selectivity and specificity for sensing metal ions due to their strong affinity with the carbonyl, hydroxy, carboxyl, and amino groups, making them ideal for the sensitive and selective detection of metal ions. The fluorescence emission spectra were recorded at the excitation wavelength of 320 nm by the addition of various metal ions such as Al^3+^, Ca^2+^, Cd^2+^, Co^2+^, Cr^3+^, Cu^2+^, Fe^3+^, Hg^2+^, Mn^2+^, Ni^2+^, Pb^2+^, and Zn^2+^ with a concentration of 500 μM. As shown in Figure 6a, the Fe^3+^ ions had oblivious fluorescence quenching (a remarkable decline in the fluorescence intensity) compared with the NN-CDs with other metal ions. The spectral results suggested that the NN-CDs had excellent selectivity toward the detection of Fe^3+^ ions. In order to find the limit of sensitivity, Fe^3+^ ions with different concentrations were added to the aqueous solution of the NN-CDs and their fluorescence emissions were recorded. The fluorescence emission intensities of the aqueous solution of the NN-CDs gradually declined with the continuous addition (increasing concentration) of Fe^3+^ ions, as shown in Figure 6b. In addition, the variation in the fluorescence emission intensity (F_0_-F/F_0_) versus the concentration of Fe^3+^ ions was displayed in a Stern–Volmer plot (Figure 6c). These showed a good linearity between the fluorescence intensity and the Fe^3+^ ion concentration between 5 and 25 μM with a correlation coefficient of R^2^ = 0.997. To further evaluate its detection ability of Fe^3+^ ions in aqueous solutions, the detection limit of the NN-CDs for Fe^3+^ ions was calculated by the equation Limit of Detection (LOD) = 3δ/S, where δ and S are the standard deviation (n = 5) and slope of the linear calibration plot, respectively [8]. The LOD was estimated to be 0.86 µM, which was obviously lower than the maximum standard limit of 5.36 µM for Fe^3+^ ions in drinking water as defined by the World Health Organization (WHO) [8]. The LOD of the synthesized NN-CDs for Fe^3+^ ions was low and comparable with earlier-reported carbon nanoparticle/carbon dots; the corresponding results are shown in Table 1. Figure 6d displays the digital photographs of the aqueous suspension of the NN-CDs before and after adding Fe^3+^ ions under an illumination of 365 nm of UV light as well as the schematic representation of the fluorescence emission quenching triggered by the formation of Fe^3+^ chelation. The digital photographs demonstrated that the fluorescence emission of the NN-CDs was notably quenched (fluorescence turn-off) when Fe^3+^ ions were added into the NN-CD aqueous suspension. Various surface/edge functional groups such as carbonyl (C=O), hydroxy (–OH), carboxyl (O–C=O), and amino (N–H) of the NN-CDs coordinated with the Fe^3+^ ions [63]. The electron donors of the oxygen and nitrogen chelating groups formatted the covalent bond with the Fe^3+^ ions compared with the other metal ions due to its greater affinity [64]. During the formation of new bonds, the excited electrons on the surface of the NN-CDs migrated to the vacant (half-filled) 3D orbitals of the Fe^3+^ ions, facilitating the nonradiative recombination of the excitons (electron/hole) [65]. This process resulted in the significant fluorescence quenching (turn-off) of the coordinating Fe^3+^–NN-CD complex [2,66]. In addition, the ferromagnetic nature of the Fe^3+^ ions might also have been a key factor causing the splitting of the discrete energy levels of the NN-CDs, thus forming channels for the overlap of energy levels and intersystem crossing, resulting in a decrement of the fluorescence emission.

## 3. Conclusions

This research work clearly demonstrated the option for preparing economical, eco-friendly, and durable fluorescent NN-CDs from Chebulic Myrobalan as a versatile nanomaterial for selective and sensitive detection of ferric ions in an aqueous medium. In summary, one-pot hydrothermal heating was utilized to prepare NN-CDs; the prepared NN-CDs had excellent water dispersibility with abundant oxygen and nitrogen-containing functional groups on their surface and edges. The NN-CDs exhibited a near-spherical shape and were monodispersed with a narrow size distribution (1–4 nm) and exceptional stability. The NN-CDs displayed excitation-dependent fluorescent emissions and durable fluorescence. The fluorescent-sensing studies unanimously exposed the excellent sensing ability of the NN-CDs toward ferric ions by fluorescence quenching (turn-off) in an aqueous medium. The LOD of the ferric ions was 0.86 μM in a perfect linear range between 5 and 25 μM with a linear coefficient (R^2^) of 0.997. This successful selective and sensitive detection of ferric ions by quenching might have been due to the coordination (interaction) between the ferric ions and the hydroxyl/carboxyl groups on the surface/edges of the NN-CDs. This interaction caused a transfer of excited electrons/energy from the NN-CDs to the outer orbital of the ferric ions, which resulted in the fluorescence quenching of the NN-CDs. Moreover, these NN-CDs could have a broad application prospect in the field of sensors.

## 4. Materials and Methods

### Preparation of Fluorescent NN-CDs

**Scheme 1:**
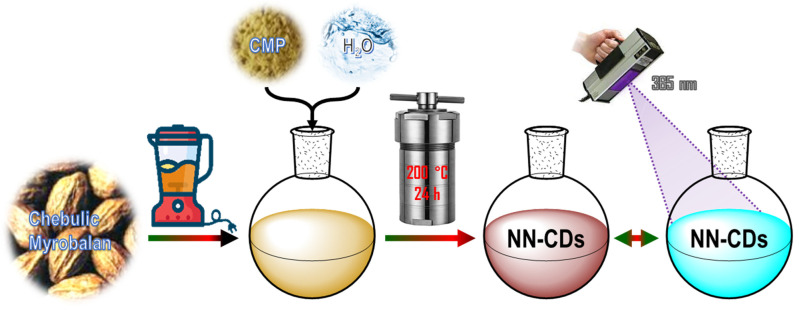
Preparation of the natural nitrogen-doped carbon dots from Chebulic Myrobalan by hydrothermal-assisted carbonization route.

Carbon dots were prepared using the hydrothermal-assisted carbonization of Chebulic Myrobalan. In a typical synthesis, the dried Chebulic Myrobalan fruit were well-ground using a commercial mixer and grinder to obtain a fine powder. Subsequently, 500 mg of Chebulic Myrobalan powder (CMP) was dispersed in 50 mL of double-distilled water. The turbid CMP-dispersed solution was then subjected to a hydrothermal treatment for 24 h at 200 °C using a 100 mL Teflon-lined stainless steel autoclave in an oven. After the desired time, the autoclave was cooled down to room temperature. The final product of the carbon dots was recovered from the reaction mixture via simple filtration using a 0.22 µm microporous mixed cellulose ester membrane filter. The resulting carbon dot solution was dried in a vacuum freeze dryer to obtain carbon dots in a powder form and these were denoted as natural nitrogen-doped carbon dots (NN-CDs). The preparation of the NN-CDs is briefly displayed in Scheme 1. Eventually, the obtained NN-CDs were examined by various physicochemical analytical techniques.

## Figures and Tables

**Table 1:** Comparison of the fluorescent sensors for the ideal sensing of ferric ions in an aqueous medium.

No.	Carbon Precursor	Excitation (nm)	QY (%)	LOD (μM)	Reference
1	PPD/quinol	340	--	0.53	[5]
2	*B. flabellifer*	310	19.4	2.01	[6]
3	*P. acidus*	350	14.0	0.9	[63]
4	Graphite	365	11.2	1.8	[67]
5	Crop biomass	380	3.5	5.23	[35]
6	Wolfberry	350	22	3.0	[10]
7	Sulfanilic acid	400	--	2.55	[68]
8	*P. avium*	310	13	0.96	[65]
9	Citric acid	350	36.8	1.4	[25]
10	Tween^®^80	350	75.5	6.5	[27]
11	L-glutamic acid	360	12.5	3.8	[29]
12	Lignin	340	23.68	0.77	[41]
13	Chebulic Myrobalan	320	15	0.86	This work

## Data Availability

Not applicable.

## Supplementary Materials

The following supporting information can be downloaded at: https://www.mdpi.com/article/10.3390/s23020787/s1: Materials, instrumentation methods, quantum yield measurement, photobleaching measurement, and sensing of metal ions of the prepared NN-CDs. Figure S1: Energy-dispersive X-ray spectrum of the prepared NN-CDs; Figure S2: ATR-FTIR spectrum of the prepared NN-CDs; Figure S3: (a) Fluorescence excitation and emission spectra of the prepared NN-CDs; (b) fluorescence excitation-dependent emission normalized spectra of the prepared NN-CDs; Figure S4: (a) Fluorescence emission spectra of the prepared aqueous solution of NN-CDs before and after 365 nm UV light continuous irradiation (inset: digital photographs of the aqueous solution of NN-CDs before and after 365 nm UV light continuous irradiation).
