# Supplementary material for: Natural Nitrogen-Doped Carbon Dots Obtained from Hydrothermal Carbonization of Chebulic Myrobalan and Their Sensing Ability toward Heavy Metal Ions
Source: Sensors (Basel). 2023 Jan 10;23(2):787. doi: 10.3390/s23020787 (PMC9865267; doi:10.3390/s23020787)
Supplement: Supplementary file 1 [file sensors-23-00787-s001.zip › sensors-2073422-supplementary.pdf]

Supplementary Materials

Materials

Chebulic Myrobalan fruits were procured from Tamil Nadu, India. The analytical grade of metal salts such as $AlCl_3$, $CaCl_2$, $Cd(CH_3OO)_2$, $Co(OOCH_3)_2$, $CrCl_3$, $CuCl_2$, $FeCl_3$, $HgCl_2$, $MnSO_4$, $NiCl_2$, $Pb(NO_3)_2$, and $ZnCl_2$ was purchased from Ducksan chemicals, Republic of Korea. Quinine sulfate and sulfuric acid (H_2SO_4) were purchased from Sigma-Aldrich, Republic of Korea. All the chemicals were used as received, and the double distilled water was used throughout the experiments.

Instrumentation Methods

Natural nitrogen-doped carbon dots (NN-CDs) were synthesized from dried Chebulic Myrobalan fruits by simple hydrothermal method. The obtained NN-CDs were thoroughly characterized by various physicochemical techniques such as X-ray diffraction (XRD), Raman spectroscopy, Attenuated total reflectance-Fourier transform infrared (ATR-FTIR) spectroscopy, X-ray photoelectron spectroscopy (XPS), field emission scanning electron microscopy (FESEM) with energy-dispersive X-ray (EDX) spectroscopy, High-resolution transmittance electron microscopy (HRTEM), Ultraviolet-visible (UV-vis) absorption spectroscopy, and fluorescence spectroscopy. The XRD measurements were carried out using a PANalytical X'Pert^3 MRD diffractometer with monochromatized Cu Kα radiation (λ = 1.54 Å) at 40 kV and 30 mA and were recorded in the range from 10 to 80° (2θ). Raman spectrum was recorded on the XploRA Micro-Raman spectrophotometer (Horiba) with the range between 50 and 4000 cm^{-1} at the core research support center for natural products and medical materials of Yeungnam University. ATR-FTIR spectra were recorded in transmittance mode on a Perkin Elmer Spectrum Two in the wavenumber range from 400 to 4000 cm^{-1} by the addition of 8 scans at a resolution of 8 cm^{-1}. XPS spectra were achieved using a K-Alpha (Thermo Scientific). CasaXPS software was used for the deconvolution of the high-resolution XPS spectra. FESEM with EDX spectral analysis was carried out on a Hitachi S-4800 equipped with EDX at an accelerating voltage of 10/15 kV. HRTEM images were performed with an FEI-Tecnai TF-20 transmission electron microscope with an operating accelerating voltage of 200 kV. UV-vis absorption spectra were recorded from 200 to 700 nm using an OPTIZEN 3220UV spectrophotometer. Excitation and emission fluorescence spectra were recorded using a Hitachi F-7000 fluorescence spectrophotometer. The excitation wavelength was varied to determine the maximum emission intensity was achieved by varying the excitation wavelength. The slit width was fixed at 5 nm, and the scan speed was set to 400 nm/min.

Quantum Yield Measurement of the Prepared NN-CDs

The quantum yield (QY) of the synthesized NN-CDs was calculated by using quinine sulfate in 0.1 M H_2SO_4 (QY_R is 0.54) as a standard reference and was calculated using the following equation (1):

Quantum yield (QY) = $QY_R \dfrac{I_S A_R (n_S)^2}{I_R A_S (n_R)^2}$ (S1)

where, "I" is the measured integrated fluorescent emission intensity, "n" is the refractive index of the solvent, and "A" is the absorbance (intensity). The subscript "R" and "S" refer to the known fluorescent reference and standard for the synthesized sample, respectively.

Photobleaching Measurements of the Prepared NN-CDs

The photobleaching stability of the synthesized NN-CDs was examined under UV light (365 nm) continuous irradiation for 100 min. The fluorescence intensity of the NN-CDs aqueous solution was measured before and after UV-light irradiation.

Sensing of Metal Ions Using the Prepared NN-CDs

Sensing of Fe^{3+} ion was performed using a 1 cm path length quartz cell at room temperature with a fluorescence excitation wavelength of 320 nm. The fluorescence emission spectra for the mixture of NN-CDs (0.5 mL) and double distilled water (0.5 mL) were measured as a blank. The selectivity for Fe^{3+} sensing of NN-CDs was confirmed by adding 0.5 mL of twelve different common metal ions solutions (Al^{3+}, Ca^{2+}, Cd^{2+}, Co^{2+}, Cr^{3+}, Cu^{2+}, Fe^{3+}, Hg^{2+}, Mn^{2+}, Ni^{2+}, $Pb^{2+,}$ and Zn^{2+} ions) with a concentration of 1 mM to 0.5 mL of the NN-CDs aqueous solution. The fluorescence emission spectra were recorded after reaction for 20 seconds at room temperature by adopting the same procedure as that of a blank sample. Then, the sensitivity of Fe^{3+} ion in the presence of NN-CDs was conducted as follows; 0.5 mL of NN-CDs was taken in the quartz cell, followed by the addition of 0.5 mL of various concentrations of Fe^{3+} ion (5–25 μM), the fluorescence intensity was recorded after 20 seconds of interaction time.

Structural Characterizations of the Prepared NN-CDs

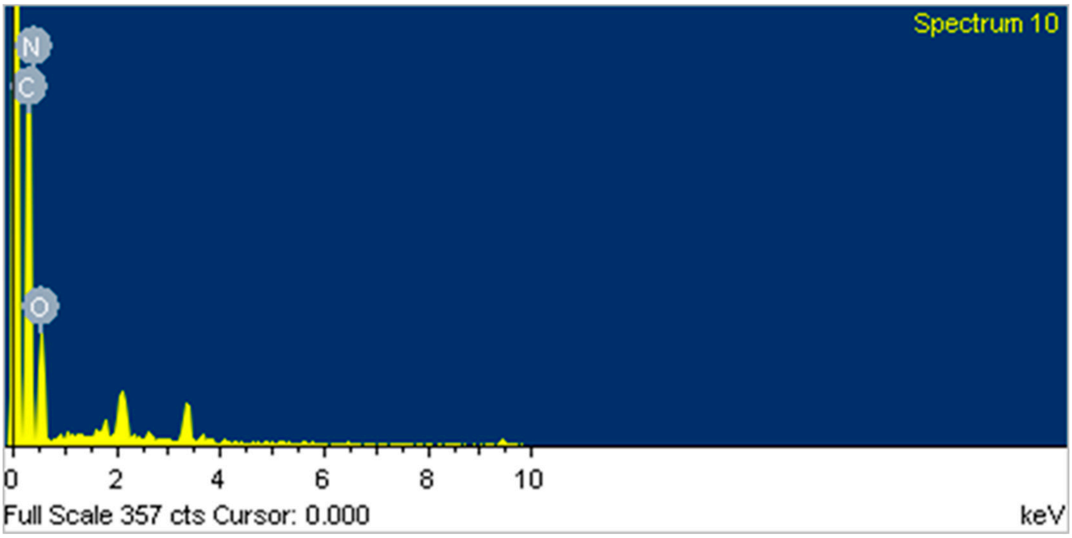

Figure S1. Energy-dispersive X-ray spectrum of the prepared NN-CDs.

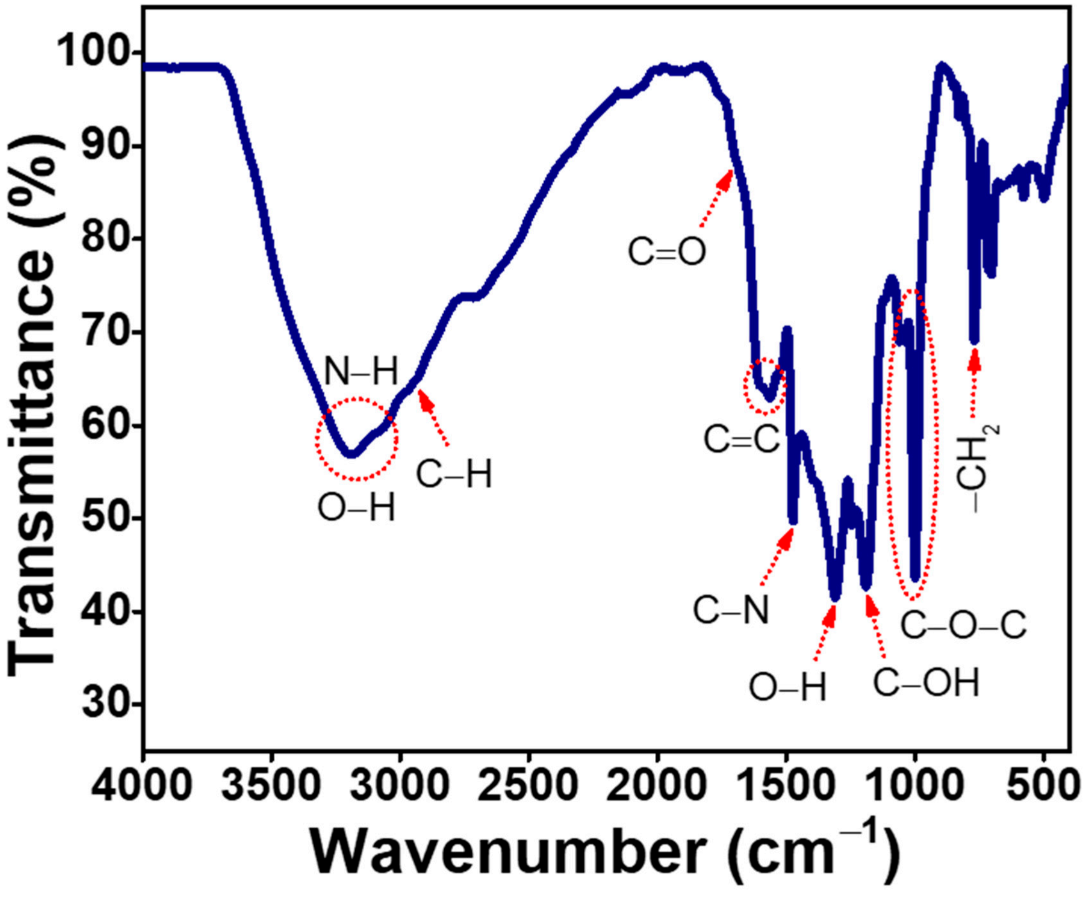

Figure S2. ATR-FTIR spectrum of the prepared NN-CDs.

Optical Characterizations of the Prepared NN-CDs

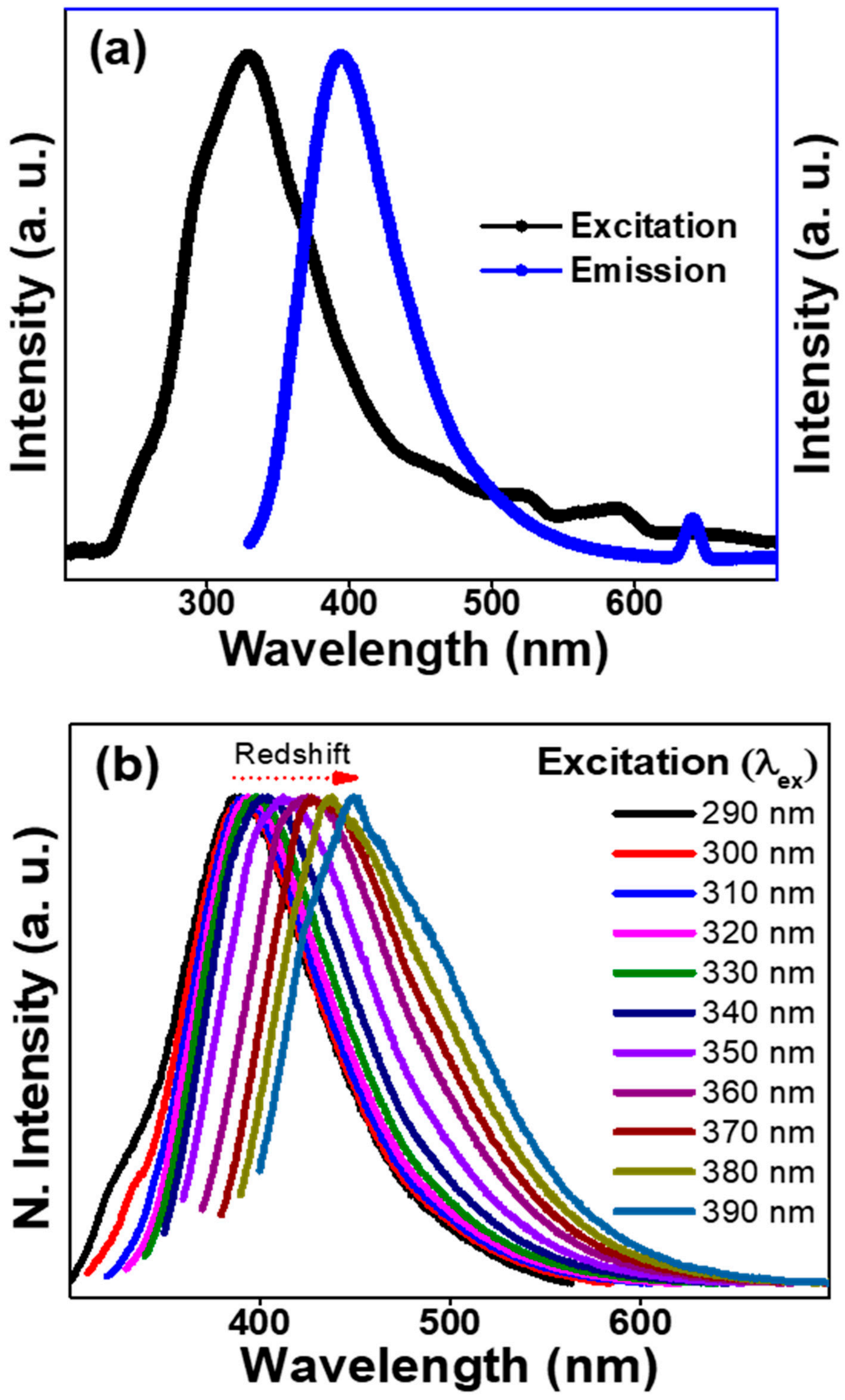

Figure S3. (a) Fluorescence excitation and emission spectra of the prepared NN-CDs; (b) Fluorescence excitation-dependent emission normalized spectra of the prepared NN-CDs.

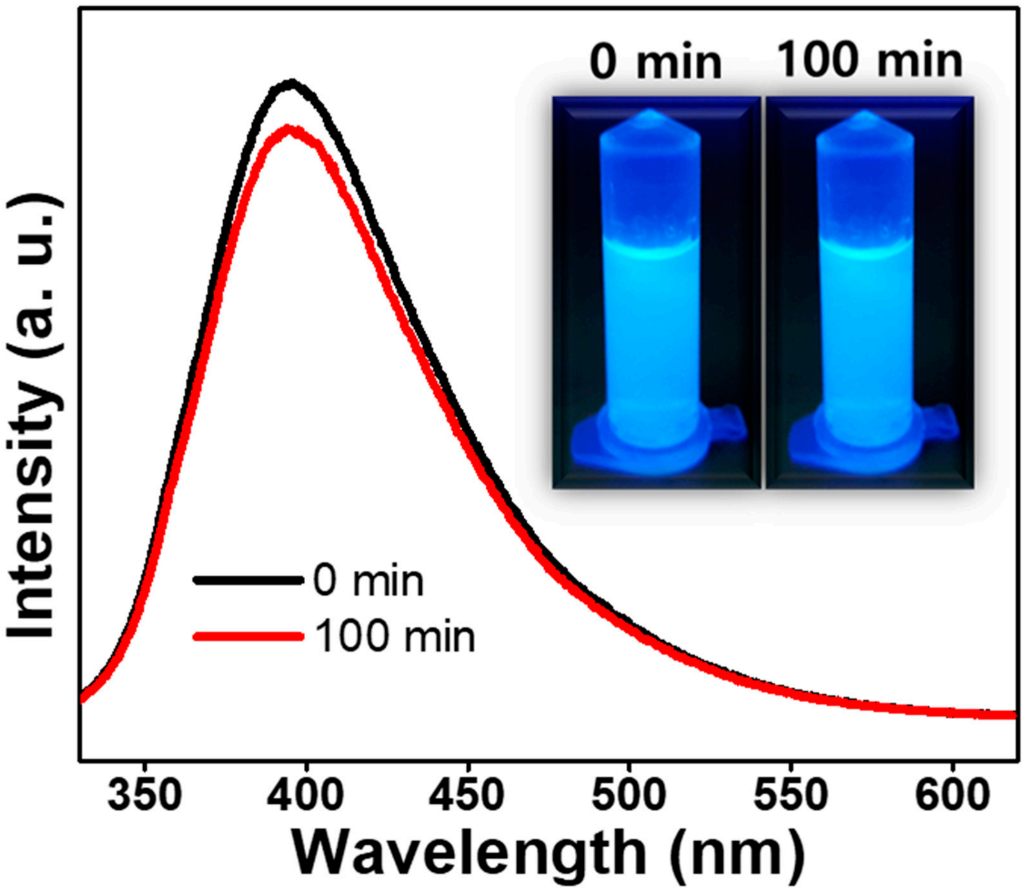

Figure S4. (a) Fluorescence emission spectra of the prepared NN-CDs aqueous solution before and after 365 nm UV light continuous irradiation (Inset: Digital photographs of NN-CDs aqueous solution before and after 365 nm UV light continuous irradiation under UV light).
